# New Appliances and Things Medical

**Published:** 1901-01-19

**Authors:** 


### NEW APPLIANCES AND THINGS MEDICAL.

[We shall be glad to receive, at our Office, 28 & 29 Southampton Street, Strand, London, W.C., from the manufacturers, specimens of all new preparations and appliances which may be brought out from time to time.]

### INVALID STOUT.

(COLEMAN'S CROWN IMPERIAL.) AGENTS: NEWTON AND LAWRENCE, QUEEN STREET, NORWICH.

This stout appears to be a beverage of more than ordinary merits for the nourishment of debilitated invalids. It is pleasant to the taste, of good alcoholic strength, and, as far as we can detect without elaborate analysis, of commendable purity. For all those invalids who require a stimulant of this nature, which combines to a certain extent nutritive food properties, we commend this particular brand.

### DR. ENOCH'S LIQUEUR IRONOL.

(SOLE AGENT: HANS HOFF, 29 NEW BRIDGE STREET, LONDON, E.C.)

This preparation of iron has a distinct advantage over many other similar preparations in that it is by no means unpalatable. It is not noticeably astringent, and the iron which it contains is claimed to assimilate to the extent of 80 per cent.

